# Movement patterns of large juvenile loggerhead turtles in the Mediterranean Sea: Ontogenetic space use in a small ocean basin

**DOI:** 10.1002/ece3.6370

**Published:** 2020-07-02

**Authors:** Marianna Chimienti, Monica F. Blasi, Sandra Hochscheid

**Affiliations:** ^1^ Department of Bioscience - Arctic Ecosystem Ecology Aarhus University Roskilde Denmark; ^2^ Filicudi WildLife Conservation Stimpagnato Filicudi Lipari Italia Italy; ^3^ Stazione Zoologica Anton Dohrn Marine Turtle Research Center Portici Italy

**Keywords:** foraging, hidden Markov models, life history, marine megafauna, ocean management, ontogenetic habitat use, satellite tracking, spatial ecology

## Abstract

Mechanisms that determine how, where, and when ontogenetic habitat shifts occur are mostly unknown in wild populations. Differences in size and environmental characteristics of ontogenetic habitats can lead to differences in movement patterns, behavior, habitat use, and spatial distributions across individuals of the same species. Knowledge of juvenile loggerhead turtles' dispersal, movements, and habitat use is largely unknown, especially in the Mediterranean Sea. Satellite relay data loggers were used to monitor movements, diving behavior, and water temperature of eleven large juvenile loggerhead turtles (*Caretta caretta*) deliberately caught in an oceanic habitat in the Mediterranean Sea. Hidden Markov models were used over 4,430 spatial locations to quantify the different activities performed by each individual: transit, low‐, and high‐intensity diving. Model results were then analyzed in relation to water temperature, bathymetry, and distance to the coast. The hidden Markov model differentiated between bouts of area‐restricted search as low‐ and high‐intensity diving, and transit movements. The turtles foraged in deep oceanic waters within 60 km from the coast as well as above 140 km from the coast. They used an average area of 194,802 km^2^, where most individuals used the deepest part of the Southern Tyrrhenian Sea with the highest seamounts, while only two switched to neritic foraging showing plasticity in foraging strategies among turtles of similar age classes. The foraging distribution of large juvenile loggerhead turtles, including some which were of the minimum size of adults, in the Tyrrhenian Sea is mainly concentrated in a relatively small oceanic area with predictable mesoscale oceanographic features, despite the proximity of suitable neritic foraging habitats. Our study highlights the importance of collecting high‐resolution data about species distribution and behavior across different spatio‐temporal scales and life stages for implementing conservation and dynamic ocean management actions.

## Graphical abstract

The study reveals the behavior and habitat use of large juvenile and adult‐sized loggerhead turtles in a spatially confined oceanic foraging area in the Western Mediterranean Sea. Hidden Markov models were used to quantify different behavioral states along the movement paths of satellite‐tracked turtles. Most turtles foraged in deep oceanic waters of the deepest part of the Southern Tyrrhenian Sea with the highest seamounts, while few individuals switched to neritic foraging showing plasticity in foraging strategies among turtles of similar life stages.

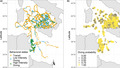

## 1 INTRODUCTION

Animals that are capable of locomotion disperse in the environment in search for essential resources, which leads to various distribution patterns over small‐ to large‐scale geographic ranges. Knowing when and where to find a species of interest, especially when it is impacted by human activities, is fundamental to effective conservation management. The spatial distribution of animals often also varies over time, such as, for example, in species with ontogenetic shifts in habitat use or with seasonal segregation of habitats (e.g., Alerstam, Hedenström, & Åkesson, 2003; Andrews‐Goff et al., 2018; Matich & Heithaus, 2015). Studying how wild animals move and disperse in their natural environment presents challenges, as direct observations are often difficult or impossible. Over the past few decades, the rapid development of miniaturized animal‐borne tags has made it possible to record movements of wild animals, aspects of their behavior and physiology, and properties of their environments (Hussey et al., 2015; Kays, Crofoot, Jetz, & Wikelski, 2015). These technological advances have allowed researchers to address key ecological and physiological questions about what animals do along their movement trajectories, costs/benefits of different movement patterns, prey pursuit, interaction with conspecifics and surrounding habitat, and how they manage their time and energy budgets (Amélineau et al., 2018; Flack, Nagy, Fiedler, Couzin, & Wikelski, 2018; Goldbogen et al., 2015).

Particularly, the marine environment is a highly dynamic system, and over the past decades, marine management and policy started to evolve toward solutions that consider ecosystems in their entirety (Maxwell et al., 2015; Scales et al., 2017). Understanding of animal movement patterns and spatial distributions, biophysical mechanisms regulating predator–prey dynamics, as well as the growing use of marine resources (shipping, fishing and marine renewables), is imperative to implement conservation management strategies effectively (Maxwell et al., 2015; Patterson et al., 2016). Being equipped with novel bio‐logging sensors, as cameras, radars, salinity, and temperature sensors, marine megafauna (seabirds, marine mammals, sea turtles, sharks, and large fish) are sentinels of the marine ecosystem, providing valuable information about environmental conditions encountered and human activities (Fedak, 2004; Hays et al., 2016; Mallett et al., 2018; Weimerskirch et al., 2020.

A number of recent publications have highlighted how bio‐logging studies, including satellite tracking, have advanced our knowledge on marine megafauna and, in particular, on sea turtles (Godley et al., 2008; Hays et al., 2016; Hays & Hawkes, 2018; Jeffers & Godley, 2016). Nonetheless, because of their elusive nature, sea turtles retain some mysteries yet to be discovered, which is not surprising considering that complex life cycles including a succession of life stages and corresponding ontogenetic habitat shifts are characteristic of each species. The most common life history pattern is characterized by the oceanic–neritic developmental model, for which the best‐known example is the Atlantic population of loggerhead turtles (*Caretta caretta*) (Bolten, 2003). It was demonstrated that hatchlings, once they have entered the sea, swim innately toward the open sea until they are caught by the great north Atlantic gyre and dispersed over the entire ocean basin, where they spent between 7 and 12 years of feeding in the epipelagic zone (oceanic juvenile stage). After a transitional phase, turtles then recruit to benthic foraging habitats where the neritic juvenile stage begins. After the neritic juveniles have grown into adult size and begin to reproduce, they conduct regular migrations between foraging areas and reproductive areas close to their natal site, showing usually high fidelity to both areas (Broderick, Coyne, Fuller, Glen, & Godley, 2007; Schofield et al., 2010; Tucker, MacDonald, & Seminoff, 2014). The reasons and underlying mechanisms for these ontogenetic habitat shifts are mostly unknown, and research is further complicated by recent findings that these shifts may be facultative and even reversible. Indeed, satellite‐tracking studies in the Atlantic have shown a dichotomy in habitat use by large juvenile and adult loggerhead turtles suggesting a high plasticity in foraging and migratory strategies (Hawkes et al., 2006; Mansfield, Saba, Keinath, & Musick, 2009).

The Mediterranean Sea, in contrast to the huge ocean basins of the Atlantic or the Pacific, is a comparatively small home to sea turtles comprising <1% of the world ocean area, and because of its distinct geographic, oceanographic, and biological characteristics, the life history traits of the local loggerhead turtles may vary from the Atlantic model. In fact, the relatively small water body (2,967,000 km^2^), its division in two basins that communicate through physical bottlenecks, and the much higher proportion of the neritic zone, inevitably brings oceanic stage juveniles in the proximity of coasts, which they may leave again after unknown periods to return to the oceanic zone. This may blur the orthodox partition of the developmental stages and lead to differences in behavior, habitat use, and spatial distribution.

Satellite tracking has also been intensively used in the Mediterranean to identify migratory corridors of adult sea turtles, important feeding areas, and spatial distribution in some oceanic areas, as reviewed by Luschi and Casale (2014). More recently, Jeffers and Godley (2016) have shown by analyzing 369 scientific papers and questionnaires completed by 171 experts that approximately 13% of the worldwide tracking on sea turtles has been conducted in the Mediterranean region; however, important knowledge gaps remain and there is a need to focus future tracking effort on those key questions that still require answers. Among the top ten research priorities, Mediterranean sea turtle experts have called for satellite telemetry studies to assess movement patterns of juvenile turtles and to identify important oceanic foraging areas (Casale et al., 2018). In particular, for loggerhead turtles such research effort should be carried out in the Ligurian Sea, Tyrrhenian Sea, Ionian Sea, and Sicily Channel, which were indicated as data deficient areas. In addition, the Demographic Working Group, which was created during the 5th Mediterranean Conference on Sea Turtles in 2015 (Dalaman, Turkey) and consists of 14 experts from the region, recommended “that future studies using satellite telemetry should make an effort of capturing healthy individuals directly from the marine habitats which are the focus of the study” (Demographic Working Group, 2015). This stems from the realization that the current knowledge on spatio‐temporal movement patterns of Mediterranean juveniles is based almost exclusively on rehabilitated turtles or individuals that were accidentally caught by fishing gear (Cardona, Fernández, Revelles, & Aguilar, 2012; Cardona et al., 2009; Luschi & Casale, 2014). Either way, there is a possibility that the movement patterns displayed by these turtles may have been biased by time spent in confined spaces and maintenance conditions in rehabilitation centers or by trauma, stress, and injuries inflicted during fishing operations or other human activities (Cardona et al., 2012).

Recent studies on rehabilitated and wild captured loggerhead turtles in the Western Mediterranean Sea indicated the presence of an important foraging and overwintering areas in the Tyrrhenian Sea, due to volcanic islands and seamounts, which comprise extensive neritic and oceanic habitats within short distances (Blasi & Mattei, 2017; Luschi, Mencacci, Cerritelli, Papetti, & Hochscheid, 2018). Encouraged by the prospect of elucidating turtle movement patterns in a potentially important foraging area in the Mediterranean, we set out to capture juvenile turtles directly from an oceanic area around an archipelago in the south of the Tyrrhenian Sea. We monitored the movement patterns and diving behavior of these turtles through satellite relay data loggers, collecting information about both horizontal and vertical movements as well as about the surrounding aquatic environment in which the turtles moved. We aimed at characterizing the spatial distribution of loggerhead turtles within this confined oceanic area by analyzing movement patterns in relation to their location and to explore how proximity to the coast and water temperature affect their behavioral decision making.

## 2 METHODS

### 2.1 Instruments: Tags and configuration

We used satellite relay data loggers (SRDL) to track the turtles' movements and diving behavior. These tags collect data from integrated sensors at user‐defined intervals, process them onboard, and relay them via the ARGOS satellite system operated by Collecte Localisation Satellite (https://www.cls.fr/). In particular, for this study we used CTD/Fluorometer Oceanography SRDL (Sea Mammal Research Unit [SMRU] Instrumentation, Scottish Oceans Institute, University of St Andrews, St Andrews, Scotland), which incorporate many of the features of the SMRU SRDL tags plus a fluorometer for chlorophyll concentration measurements and temperature and conductivity sensors that deliver oceanographic quality temperature and salinity profiles. A complete list of the specifications of the CTD tags are given on the manufacturer's website (http://www.smru.st‐andrews.ac.uk/Instrumentation/FluorometryTag/), while here we provide details only for those features and configurations that were used in the present study.

Turtle positions were obtained through the ARGOS system: During an overpass, the satellite receives messages from the tag carried by the turtle at fixed intervals and computes the position on the basis of Doppler effect measurements. During these messages, also data on diving behavior and water temperature were transmitted and transmission times were synchronized with the time that the turtle was at the water surface through the tag's integrated saltwater switch. Diving data were derived from measurements of the pressure sensor that sampled dive depth in relation to an internal real time clock every 4 s. A dive was defined to start when the tag was submerged (determined through the saltwater switch) and below 4 m for 30 s, and ended either when the tag was above the sea surface (i.e., the saltwater switch in the dry state) or above 4 m. All dives were counted and the number of dives was transmitted, too. In addition, the saltwater switch was used to define the “haul‐out” behavior, indicating that the turtle stayed at the surface, with the carapace (and hence the satellite tag) out of water: a haul out started when the tag was “dry” for 5 min and ended when it was “wet” for 40 s. The temperature (as the other environmental sensors) was checked at 1‐s intervals during data collections for vertical profiles. Each profile contained 17 cut points (a temperature value at a given depth and time), consisting of one at the minimum depth and one at the maximum depth and of 15 fixed points that are equally spaced between the minimum and maximum depths. The pressure sensor operated in the range of 0–2,000 dbar with an accuracy of 2 dbar (±[0.3 + 0.035% * reading]/°K) and a resolution of 0.05 dbar, and the temperature sensor operated in the range of −5° to 35°C with an accuracy of ±0.005°C and a resolution of 0.001°C.

### 2.2 Turtle capture and tag deployments

Eleven large juvenile loggerhead turtles (curved carapace length: 55–75 cm) were used for this study, and some (*n* = 7) were in the minimum size range of female turtles observed nesting (Casale et al., 2018), but we did not establish their state of reproductive maturity (see Table 1 for turtle sizes). Since we could not ascertain if some of the turtles were already reproductively active we assumed that all turtles, including the larger individuals, were still juveniles (Blasi & Mattei, 2017). In November 2016, July 2017, and June 2018, turtles were spotted by boat when resting at the water surface, upon which they were approached and hand‐caught with a custom‐made dip net. All turtles were caught in an approximately 500‐km^2^ area around the island of Filicudi (38.5147°N, 14.6840°E), Aeolian Archipelago, and Sicily (radius = 12.6 km), except turtle ID 165768 (see below). The turtles were then taken to the Filicudi First Aid Center, where they were kept temporarily in individual containers that were large enough for a turtle to turn 360°. The tanks were filled with seawater, which was replaced three times per day. All turtles were measured and underwent physical examination according to standard procedures (Blasi & Mattei, 2017). On the afternoon before the day of release, they were prepared for tag attachment. Details on the dates of capture and release are also given in Table 1. The turtles' carapaces were cleaned of algae and epibionts and roughened with sandpaper for better adhesion of the glue. A small quantity of marine silicone adhesive (Sikaflex^®^ 291i) was then applied to the second vertebral scute, and the tag was placed on top, slightly squeezing the glue, which was then evenly distributed around the tag. The turtles were left overnight in dry dog for the adhesive to cure, and all were released the following morning from the Pecorini beach in front of the center (38.558616°N, 14.565865°E).

**TABLE 1:** Summary data for 11 loggerhead turtles equipped with CTD SRDL tags

Turtle ID	Capture date	CCL (cm)	Sex	Deploy date	Date last location	Last location	Days at large	*N* locations
165766a	02/11/2016	70.5	m	04/11/2016	29/05/2017	Amvrakikos Gulf, Greece	207	715
165767	02/11/2016	65.5	f	04/11/2016	31/07/2017	Tunisian Plateau	270	1,857
165768	13/09/2016	64.5	n/a	13/10/2016	26/04/2017	S Tyrrhenian Sea	195	1,457
165769	02/11/2016	62	n/a	04/11/2016	09/06/2017	Cape Bon, Tunisia	217	1,896
162338	02/11/2016	59.5	n/a	04/11/2016	28/04/2017	S Tyrrhenian Sea	175	964
162341	04/06/2017	55	n/a	09/06/2017	11/07/2017	S Tyrrhenian Sea	33	136
162340	04/06/2017	59	n/a	09/06/2017	14/07/2017	Misratah Valley, Libya	36	500
162342	04/06/2017	58	n/a	09/06/2017	27/07/2017	S Tyrrhenian Sea	49	886
162339	04/06/2017	61	n/a	09/06/2017	02/07/2017	Heron Valley, S Ionian Sea	23	334
162343	04/06/2017	75	f	09/06/2017	16/07/2017	San Vito Lo Capo, Sicilia	37	476
165766b	21/06/2018	62	n/a	08/07/2018	02/11/2018	S Tyrrhenian Sea	117	1,107

Turtle ID 165768 was found floating by a finance guard patrol in the waters off Gaeta (41.1876°N, 13.5490°E), Lazio. The finance guards took the turtle onboard their vessel and transported the rescued animal to the Marine Turtle Research Center of the Stazione Zoologica Anton Dohrn, Napoli. The turtle was examined by a veterinary and kept for observation at the center, during which no health problems were detected. It was chosen for this study and equipped with a CTD SRDL following the same procedures as described for the other turtles and released from a boat into the open sea, just off the island of Capri (40.7469167°N, 14.04295°E).

### 2.3 Data analysis and model structure

Given the limited bandwidth of Argos platforms for transferring data and limited or irregular exposure to satellites due to the sea turtles' diving behavior, the location data were subject to measurement error and temporal irregularity and the auxiliary biotelemetry data were subject to missing or incomplete records. The Argos location error ellipses were oriented toward the x‐axis, with mean semi‐major axis *M* = 14,198 m (median = 4,637 m, *SD* = 41,406.38), semi‐ minor axis m = 802 m (median = 339 m, *SD* = 1,173.872), and orientation *c* = 87.64° (median = 89.00, *SD* = 30.41).

All tracks were visually inspected prior to the modeling exercise, and the first 24 hr of observations was excluded because the turtles' behavior could have been biased by postrelease stress. The dataset was manipulated and analyzed using the R package “momentuHMM,” following the method developed by McClintock (McClintock, 2017; McClintock & Michelot, 2018). The method implemented in “momentuHMM” allows to predict temporally regular locations, account for location measurement error, fit multiple imputations, and perform behavioral classification analysis using hidden Markov models (HMMs) for more than two behavioral states and has the ability to incorporate spatio‐temporal environmental or individual covariates on parameters (McClintock & Michelot, 2018). The function *crawlWrap* was then used to predict temporally regular locations at 6‐hr time steps assuming a bivariate normal measurement error model (McClintock, 2017).

Both dive and environmental data were collected at different temporal resolutions with respect to the location data (see above, “Instruments: sensors and configuration”) and presented gaps in the recording. To make use of such information at a spatial level, both diving data (i.e., number of dives, maximum depth, dive time, haul‐out time) and temperature data were summarized at 6‐hr periods to match the temporally regular locations. First, a summary (mean, median, and *SD*) of each temperature profile was associated with the dive during which the temperatures were recorded. Subsequently, the data were further summarized at 6‐hr intervals to be matched to each spatial location.

The multiple imputation approach was used to account for location uncertainty by repeatedly fitting the HMM to *nSims* = 100 realizations of the position process using *MIfitHMM*. The HMM is a time series model composed of an observation process (*Z*_1_, …, *Z*_T_), in which each data stream is generated by *N* state‐dependent probability distributions, and where the unobservable (hidden) state sequence (*S*_t_ ∈ {1, …, *N*}, *t* = 1,…, *T*) is assumed to be a Markov chain. The state sequence of the Markov chain is governed by a first‐order state transition probability and an initial distribution (Zucchini & MacDonald, 2009). Sea turtles move in a 3‐dimensional space; hence, in addition to the horizontal displacement (*x* and *y* coordinates), the use of auxiliary information as diving behavior and environmental variables is fundamental for understanding their habitat utilization. Initially, both diving and temperature data were considered candidate variables to be included in the model structure. The state process of the baseline model was modeled as a function of three variables: step length (i.e., straight‐line distance between two successive locations), turning angles (i.e., angles between successive steps), and number of dives performed. Step length was modeled as *Gamma* distribution, turning angle as *von Misen* distribution and number of dives as *Poisson* distribution. Models were tested with two and three behavioral states, alternative structures, and starting parameters, including maximum dive depth, haul‐out time, mean, and median values of water temperature as additional variables. Model fitting was assessed both visually and using the Akaike's information criterion (AIC, Patterson et al. (2017)). A model with three states (*N* = 3), including the variables step length, turning angles, and number of dives performed, converged successfully and aligned with biological expectations, so only this parameterization is presented here. The three states used here broadly corresponded to state 1: transit, state 2: low‐intensity diving, and state 3: high‐intensity diving.

The residuals of the models performed were checked for violations of model assumptions in terms of residual autocorrelation and normality. Due to lower AIC in the model structure without median water temperature (see Results section), we explored as additional step the effect of the median water temperature on the probability of the animals performing one of the three behavioral states above mentioned. The “nnet” R package (Venables & Ripley, 2002) was used to model the three behavioral states as a function of the median water temperature in a multinomial logistic regression framework.

Finally, for visualization purposes and to highlight area utilization, the probabilities of being in state 2 (low‐intensity diving) and state 3 (high‐intensity diving) were merged in a general diving probability and mapped. Individual kernel densities at 75 percentile and their overlap were estimated using the R package “ctmm” (Calabrese, Fleming, & Gurarie, 2016). The activity budgets that resulted from the final HMM run were plotted against distance to the coast and bathymetry. Bathymetric profiles for the Mediterranean Sea are freely available from https://portal.emodnet‐bathymetry.eu/. Data manipulation and analysis was performed using R version 3.6.1 (R Core Team, 2019).

## 3 RESULTS

**FIGURE 1:**
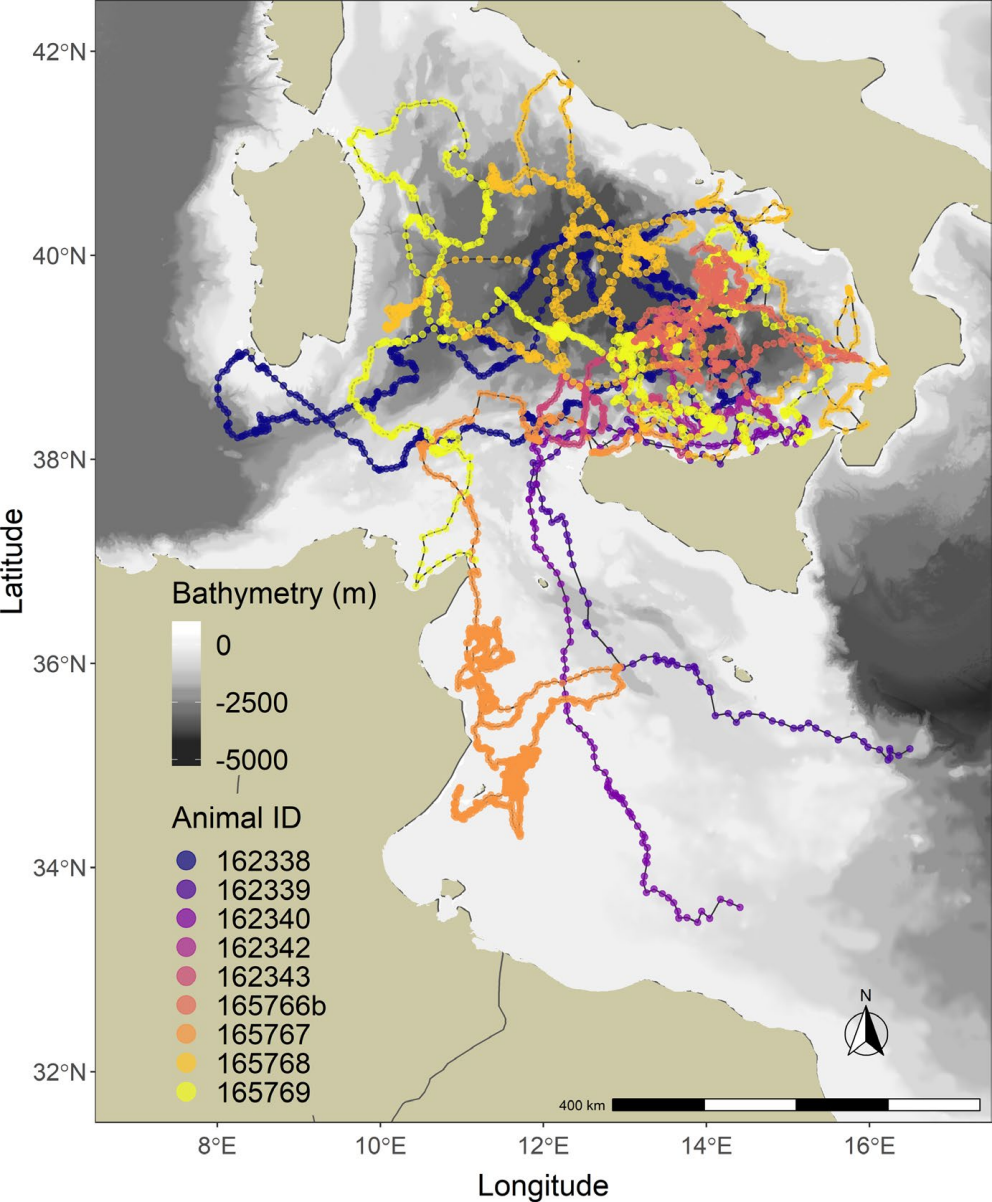
Overview of the reconstructed movements of nine juvenile and adult‐sized loggerhead turtles in the Tyrrhenian Sea

**FIGURE 2:**
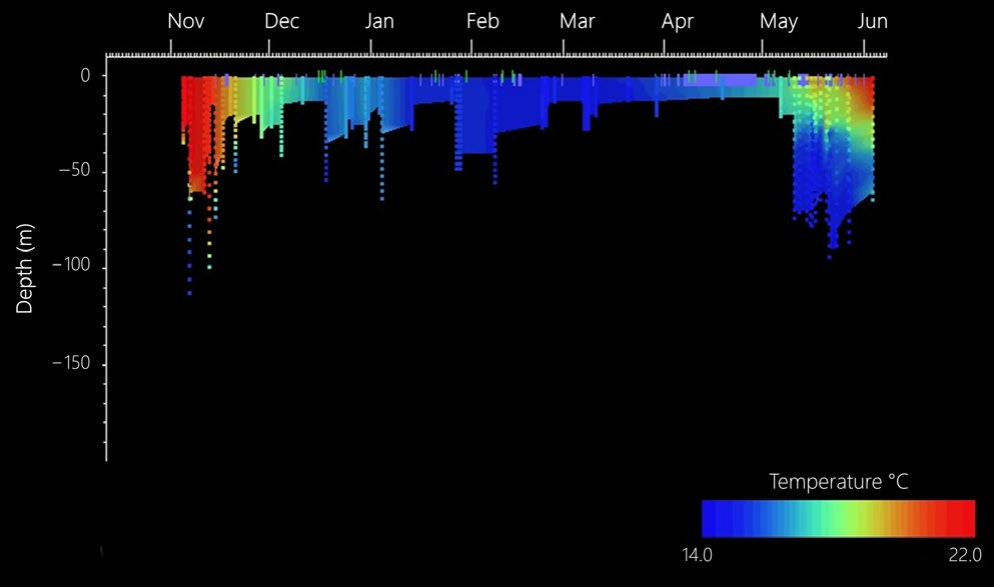
Example (from ID #162338) of a temperature range collected by the SRDL during the movements of a loggerhead turtle, both seasonal (months on *x*‐axis) and vertical (depth in [m] on the *y*‐axis)

**FIGURE 3:**
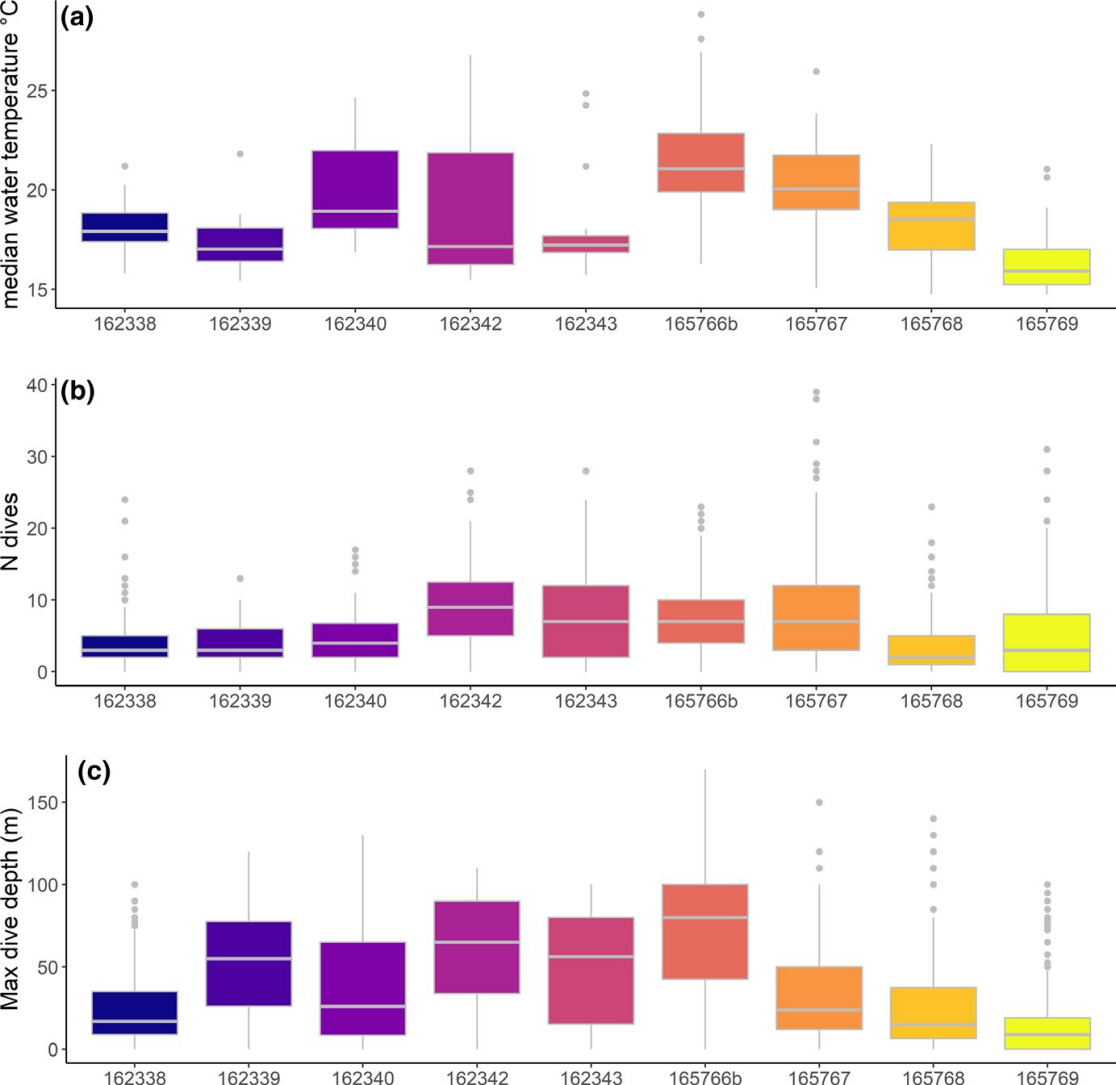
Summary of auxiliary data collected via satellite relay data loggers and used for the analysis. (a) Median water temperature (°C) recorded while diving for each animal. (b) Number of dives performed by each animal. (c) Maximum dive depth (m)

**FIGURE 4:**
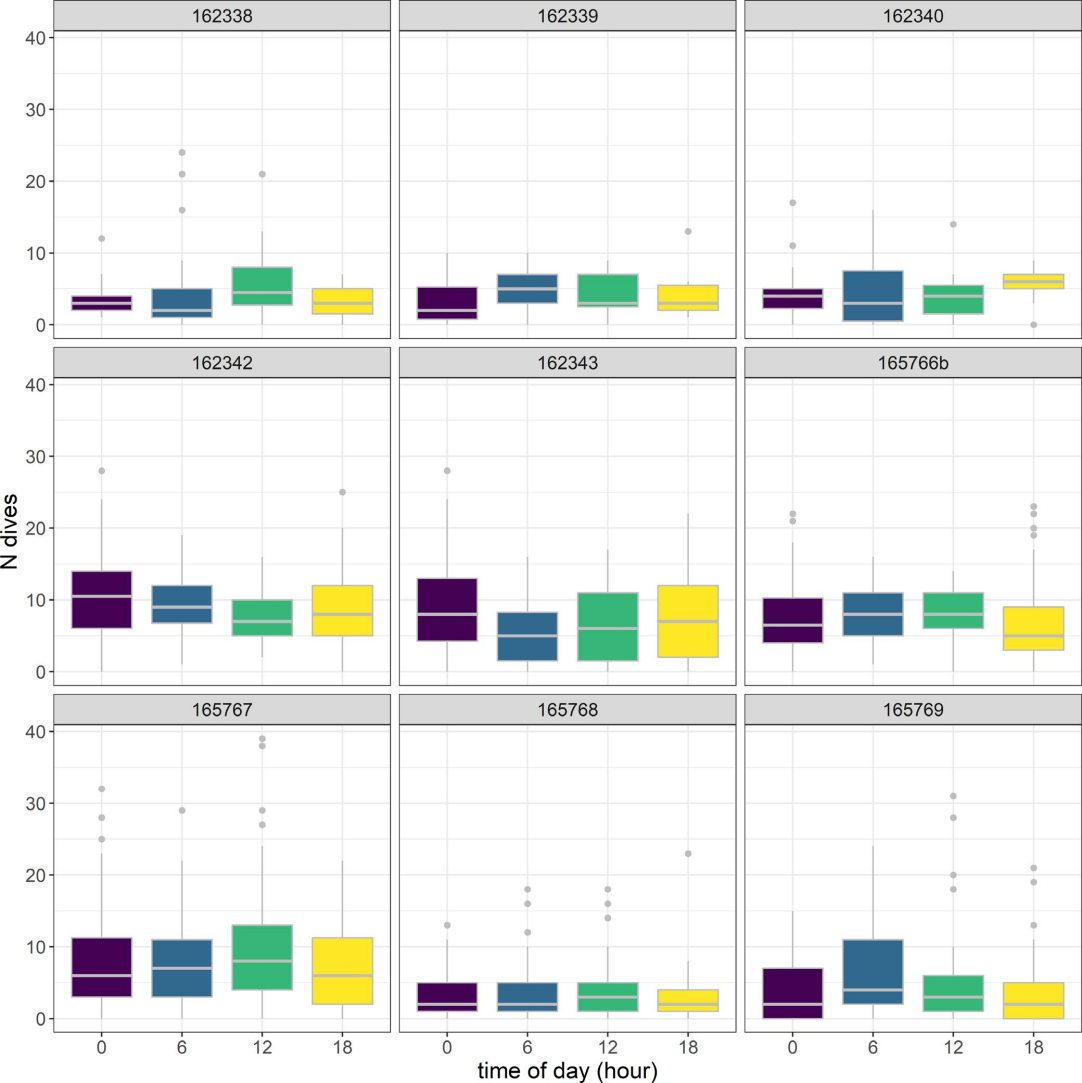
Number of dives performed by each animal every 6 hr. Shown are medians (horizontal line), interquartile ranges (upper and lower box limits), range (vertical lines), and outliers (asterisks)

Eleven juvenile loggerhead turtles were tracked over different periods, ranging from 1 month to almost 1 year (Table 1). Turtles dispersed over most of the Tyrrhenian Sea except the very northern part. Four of the eleven turtles left the Western Mediterranean Sea through the Strait of Sicily between 8 and 42 days after their release. For two of these turtles, transmissions ceased when they were in the middle of the southwestern Ionian Sea, while turtle ID's 165767 and 165766 ended up in well‐known neritic foraging habitats on the Tunisian Plateau and the Amvrakikos Gulf (Greece), respectively. Because of the poor transmitter performance and few data collected for individuals #165766a and #162341, these turtles were not included in the present analysis. The remaining nine individuals mainly roamed the Tyrrhenian Sea and the waters in the northeastern part of Tunisia (Figure 1). Turtles experienced a wide range (minimum = 14.05, maximum = 30.31°C) of water temperatures that varied both vertically and seasonally (Figures 2 and 3). Different numbers of dives were also recorded across individuals within 6‐hr periods (Figure 4; Table 2). Overall, the individuals performed 6.3 dives on average within 6‐hr periods at various depth ranges (median values ranging between 9 and 80 m) with deepest dives between 100 and 170 m (Figure 4; Table 2). All individuals made similar numbers of dives across the day (Figure 4).

**TABLE 2:** Summary of auxiliary data collected with SRDL tags for each loggerhead turtle included in the analysis

Turtle ID	Median *T*_w_ (°C)	*N* dives	Max depth (m)	Deepest dive (m)
162338	18.18 ± 1.62	4.21 ± 4.15	23.60 ± 21.50	100
162339	17.40 ± 1.81	4.109 ± 3.10	51.16 ± 33.01	120
162340	20.02 ± 2.85	4.65 ± 3.97	38.23 ± 34.78	130
162342	19.17 ± 3.74	9.21 ± 5.22	60.30 ± 31.04	110
162343	18.24 ± 2.73	7.76 ± 6.88	48.6 ± 36.0	100
165767	20.20 ± 2.11	8.419 ± 6.83	32.6 ± 27.23	150
165768	18.40 ± 2.05	3.46 ± 3.74	24.04 ± 25.19	140
165769	16.60 ± 2.13	4.63 ± 5.73	17.04 ± 22.61	100
165766b	21.50 ± 2.79	7.52 ± 4.30	74.13 ± 35.15	170

Note

Values represent means ± *SD*. *T*_w_ = water temperature, *N* dives = number of dives, Max depth = maximum depth reached during a single dive.

**FIGURE 5:**
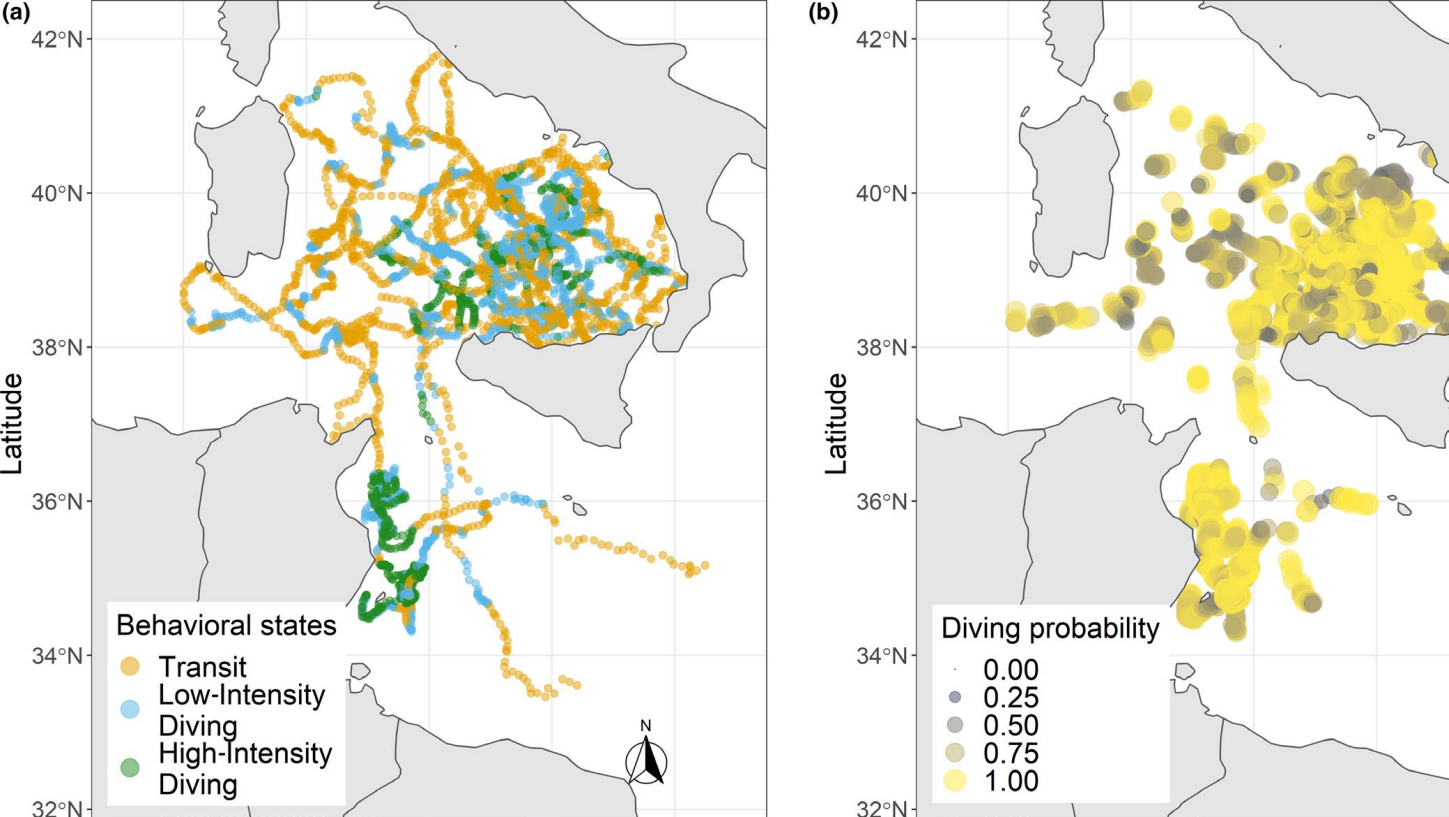
(a) Behavioral partition resulting from the hidden Markov model plotted on Argos positions for all tracked individuals. (b) Probability of diving (sum of probability of performing low‐intensity diving and high‐intensity diving)

The HMM run without median water temperature values had a lower AIC compared to the model with temperature (47,810.39 and 49,462.57, respectively). Hence, the model without water temperature was considered as the best. Under behavioral state 1 (transit, Figure 5), all individuals generally performed long step lengths (mean ± *SD*, 8.152 ± 3.27 km), kept high directional persistence toward a straight path (mean ± *SD*, 0.01 ± 1.4, radians), and performed few numbers of dives (mean ± *SD*, 1.6 ± 1.5, number of dives). Compared to state 1, under states 2 and 3, named, respectively, as low‐intensity diving and high‐intensity diving (Figure 5), all individuals performed shorter step lengths, higher variation in turning angles, and higher number of dives. Step length, turning angles, and number of dives for state 2 were estimated as (mean ± *SD*) 6.2 ± 2.0 km, 0.08 ± 1.85 radians, and 6.54 ± 2.73 number of dives, and for state 3 (mean ± *SD*), 5.3 ± 1.5 km, −0.11 ± 1.91 radians, and 14.12 ± 5.3 number of dives.

**FIGURE 6:**
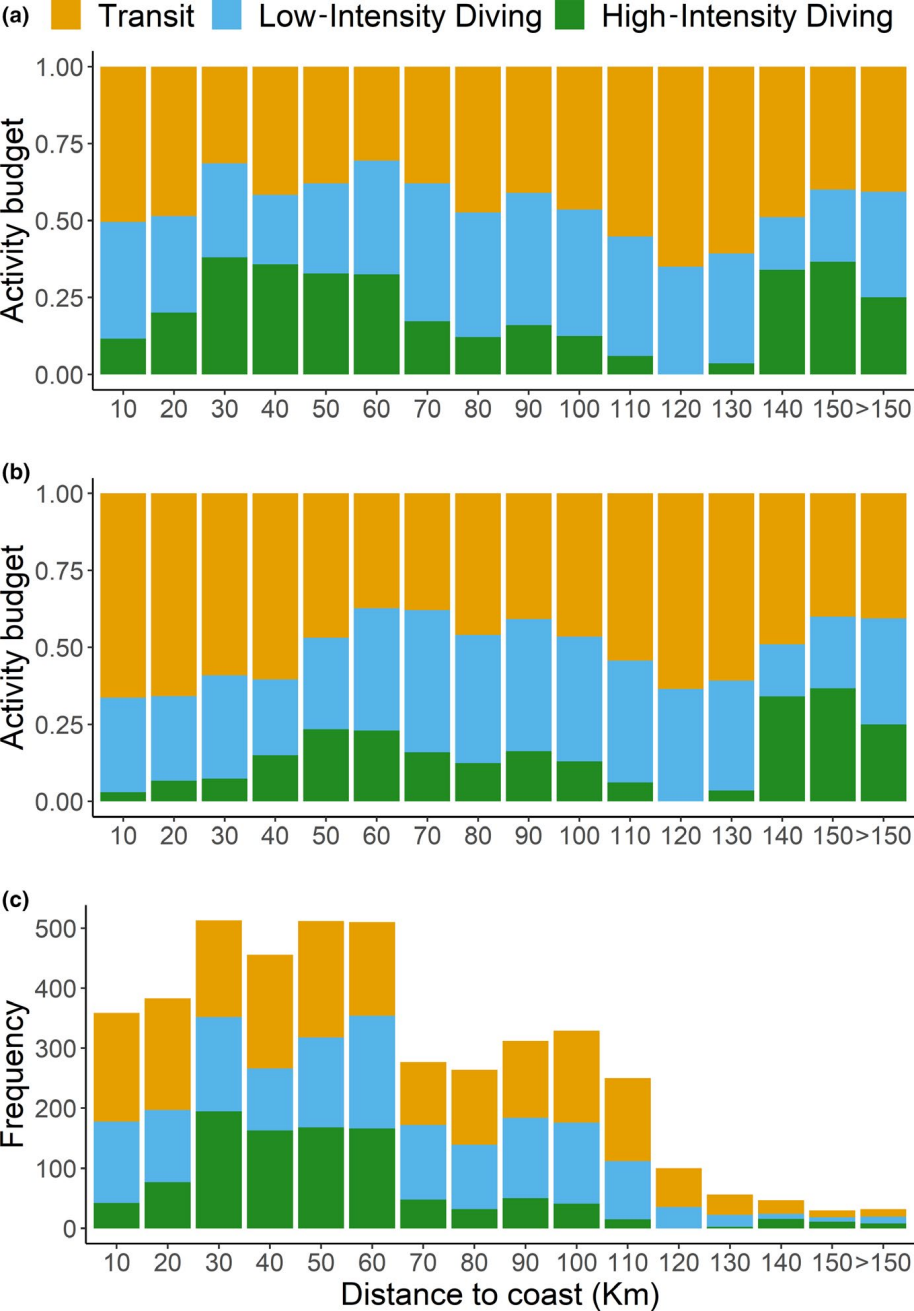
Behavioral partition resulting from the hidden Markov model in relation to the distance to the coast. (a) Plot including all individuals showing that high‐intensity diving areas concentrated between 30–60 km and 140–150 km from the coast; (b) plot including all individuals except individual #165676, as the only individual foraging on the Tunisian continental shelf. The previous high‐intensity diving area between 30 and 60 km is shifted toward 50–60 km. (c) Frequency plot of the observations showing that 50% of the observations were within 60 km from the coastline

**FIGURE 7:**
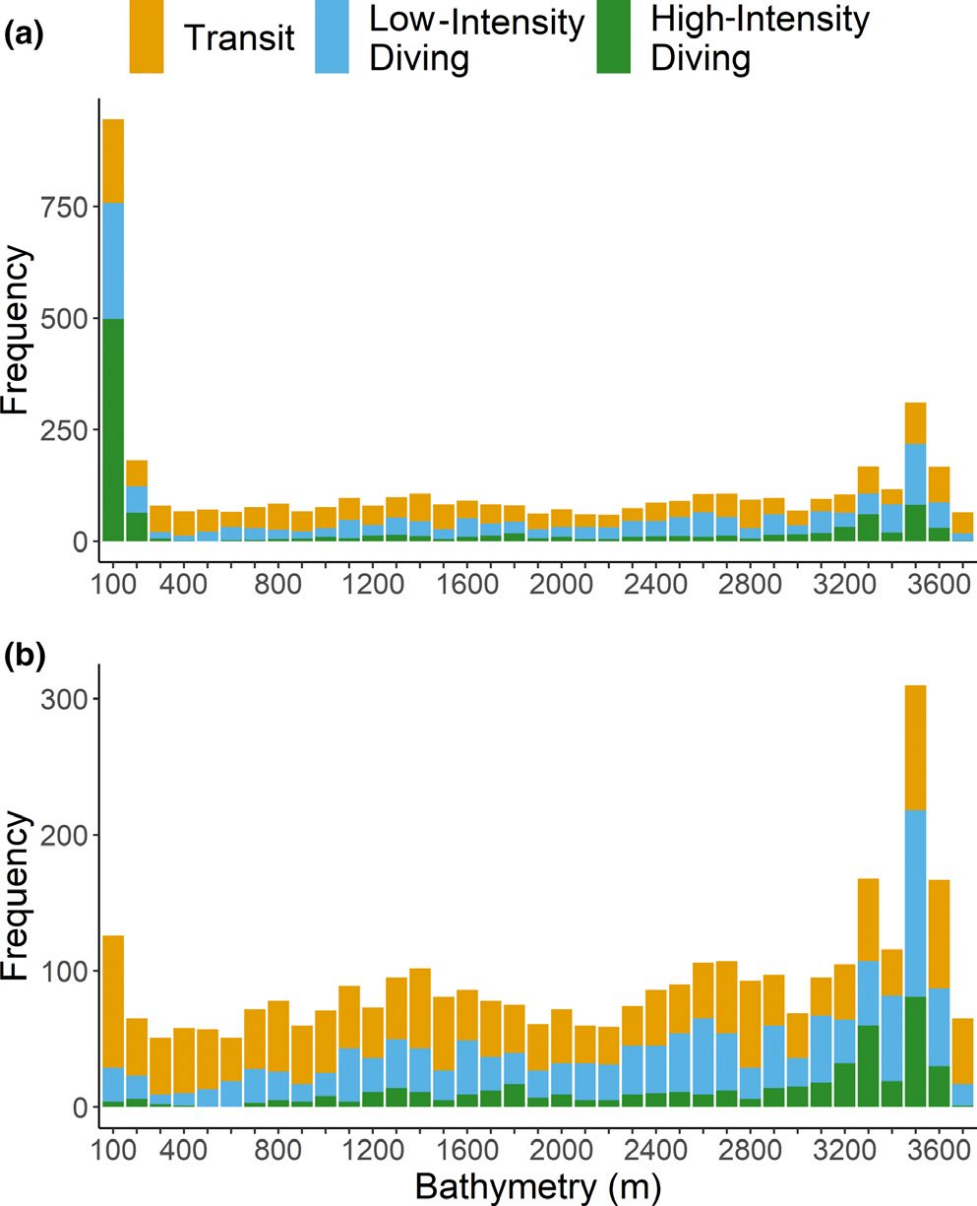
Behavioral partition resulting from the hidden Markov model in relation to the depth of the sea floor. (a) Plot including all individuals showing high frequency of activity in areas up to 100 m depth. Equal amount of activities was performed across the wide bathymetric profile with a small peak in deep waters (3,200–3,600 m). (b) Plot excluding individual #165767, hence representing all individuals using the Southern Tyrrhenian area. Individuals equally used the area characterized by a wide bathymetric profile with a peak in deep waters (3,200–3,600)

As estimated by the HMM (Figure 5), loggerhead turtles mostly used and concentrated the diving activities in the South Tyrrhenian Sea and on the Tunisian continental shelf. Individuals showed high proportions of overlap in area used (>0.5) with mean ± *SD* size of area of 194,802 ± 173,029.3 km^2^ (Table 3). High‐intensity diving activities peaked between 30–60 km and 140–150 km from the coast (Figure 6a). Individual #165767 mainly used the Tunisian continental shelf (Figure 1) performing high‐intensity activities within 30 km from the coast. By omitting this individual from the dataset, the previous peak of high‐intensity diving activity between 30–60 km is shifted to 50–60 km (Figure 6a,b), corresponding mainly to the South Tyrrhenian Sea. Fifty percent of all locations were mostly within 50 km from the coast, only 1% were farther than 150 km, and maximum distance did not exceed 190 km (Figure 6c). All individuals dispersed in areas characterized by a wide bathymetric profile (Figures 1 and 7). Highest frequency of activities occurred in waters up to 100 m deep (Figure 7a). These high activities were mainly associated with individual #165767 during its permanence on the Tunisian continental shelf. Individuals using the South Tyrrhenian Sea showed equal amount of activities across different depths, with a small peak in the deepest waters between 3,200 and 3,600 m (Figure 7b).

**TABLE 3:** Range distribution estimated via autocorrelated kernel density estimation (AKDE) for each individual track at 75% in square kilometers (km^2^) and overlap of density distributions

Turtle ID	Overlap									AKDE (km^2^)
	162338	162339	162340	162342	162343	165766b	165767	165768	165769	
162338	1.0000000	0.9506863	0.9518601	0.7240314	0.8689830	0.9049033	0.9844335	0.9983495	0.7175036	310,930.1
162339	0.9506863	1.0000000	0.9994646	0.6549347	0.8204921	0.9358546	0.9129704	0.9674953	0.6437284	434,861.1
162340	0.9518601	0.9994646	1.0000000	0.6641460	0.8108611	0.9554515	0.9071534	0.9678811	0.6572266	459,389.5
162342	0.7240314	0.6549347	0.6641460	1.0000000	0.9043530	0.5502067	0.7834689	0.7212351	0.9812998	30,227.9
162343	0.8689830	0.8204921	0.8108611	0.9043530	1.0000000	0.7674820	0.9144218	0.8413572	0.8516225	59,336.26
165766b	0.9049033	0.9358546	0.9554515	0.5502067	0.7674820	1.0000000	0.7801725	0.9087466	0.5068759	205,183.4
165767	0.9844335	0.9129704	0.9071534	0.7834689	0.9144218	0.7801725	1.0000000	0.9860428	0.7786954	188,627.6
165768	0.9983495	0.9674953	0.9678811	0.7212351	0.8413572	0.9087466	0.9860428	1.0000000	0.7305454	352,828.5
165769	0.7175036	0.6437284	0.6572266	0.9812998	0.8516225	0.5068759	0.7786954	0.7305454	1.0000000	29,379.76

When in transit state, the probability of staying in this behavior mode was 0.86, and the probability of switching to low‐intensity diving or high‐intensity diving was 0.13 and 0.01, respectively (Table 4). When performing low‐intensity diving behavior, individuals had 0.8 probability of staying in this behavior mode, with probabilities of switching to transit or high‐intensity diving of 0.13 and 0.07, respectively (Table 4). The probability of staying in high‐intensity diving state was 0.86 once the animals were performing this behavioral state. The probabilities of switching to transit or low‐intensity diving were 0.01 and 0.13, respectively (Table 4).

**TABLE 4:** Transition probability matrix resulting from the hidden Markov model run on dive and location data on nine loggerhead turtles

	Transit	Low‐intensity diving	High‐intensity diving
Transit	0.86	0.13	0.01
Low‐intensity diving	0.13	0.80	0.07
High‐intensity diving	0.01	0.13	0.86

**FIGURE 8:**
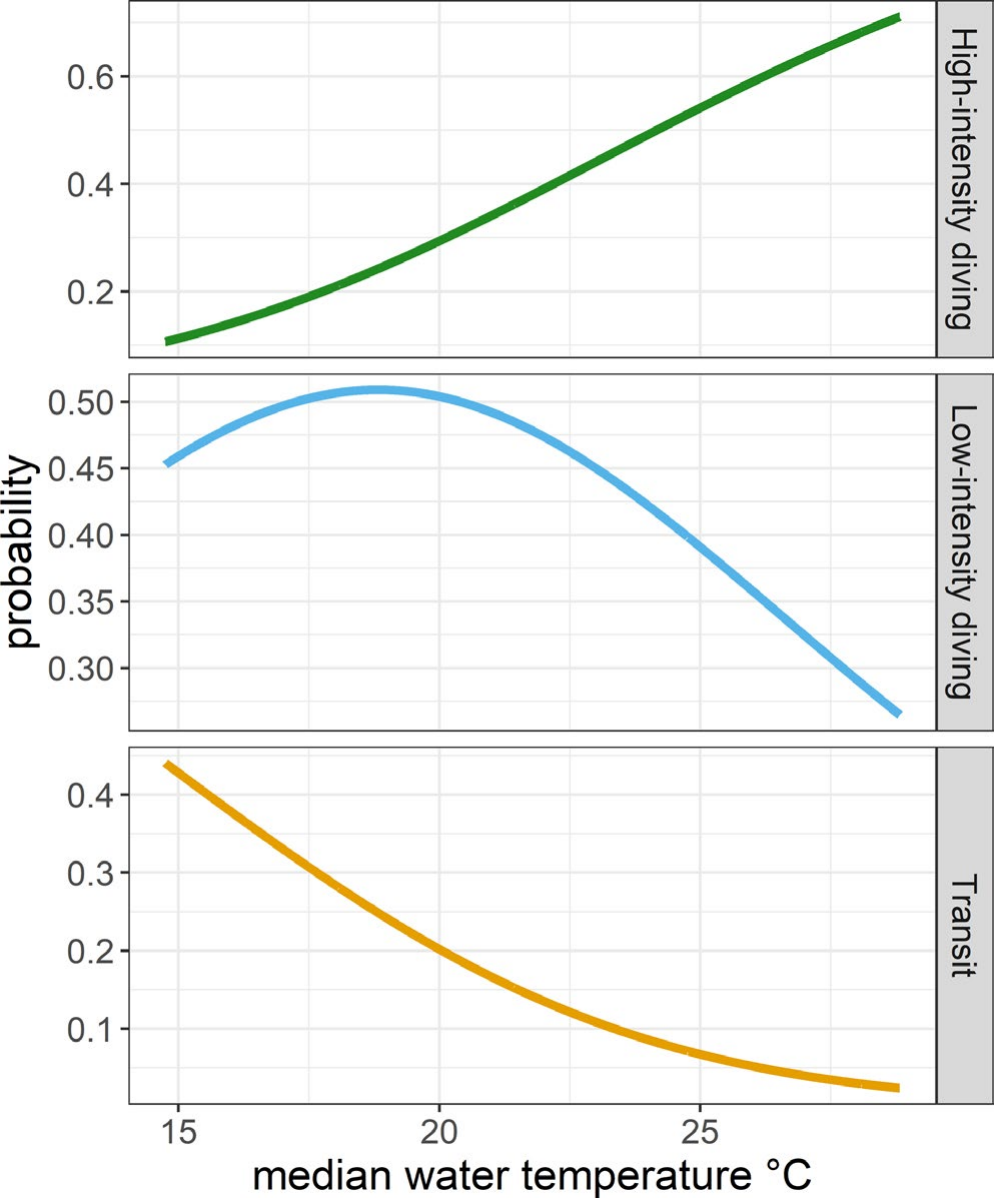
Probability of performing high‐intensity diving, low‐intensity diving, and transit behaviors in loggerhead turtles in relation to median water temperature (°C) recorded while diving

When the animals experienced higher water temperatures, the probability of being in transit behavior rapidly declined, while the probability of being in low‐intensity diving followed a slower decline (*p*‐value < .05) and the probability of performing high‐intensity diving rose (*p*‐value < .05, Figure 8).

## 4 DISCUSSION

Understanding the cryptic lives of wide‐ranging marine species throughout different life stages can be challenging. Mechanisms underlying the ontogenetic habitat shifts in loggerhead sea turtles, how and if they differ between large ocean basins and smaller seas, the location of areas most used and types of movements performed during this stage, are mostly unknown. In particular, knowledge of juvenile loggerhead turtles' dispersal, movements, and habitat use is largely unknown in the Mediterranean Sea (Casale et al., 2018).

Use of bio‐logging devices makes it possible to follow animals into remote ocean areas and collect detailed information about their behavior, physiology and surrounding environment, challenging researchers in data management, visualization, integration, and analysis. Revolutionary improvements were made over the last few years with the development of novel analytical tools such as machine learning approaches and hidden Markov models (Chimienti et al., 2016; Langrock et al., 2012; Leos‐Barajas et al., 2017; Wilson et al., 2016). Using sophisticated bio‐logging technologies and state‐of‐art statistical approaches, we quantified movements, behavior, and areas used by juvenile loggerhead turtles in the Western Mediterranean Sea.

Juvenile loggerhead turtles typically displayed hierarchical movement patterns performing “area‐restricted search” (ARS, Fauchald, Erikstad, and Skarsfjord (2000)) movements and high numbers of dives in the southern Tyrrhenian Sea and in proximity of the Tunisian continental shelf (Figure 5). When performing ARS movements, animals usually reduce movement speed and/or increase sinuosity in response to a highly clumped resource distribution (Bailleul, Lesage, & Hammill, 2010; Barraquand & Benhamou, 2008). In both low‐intensity and high‐intensity diving states, all turtles performed shorter movements with increased tortuosity and higher number of dives.

Diving activities were high in areas characterized by highly variable bathymetric profiles, both shallow as the Tunisian continental shelf and deep as the Tyrrhenian Sea (Figure 1). During the juvenile stage, individuals mainly feed on gelatinous zooplankton in oceanic habitats (water depths > 200 m), while when recruiting to neritic habitats (depths < 200 m) they switch to a diet of benthic invertebrates such as molluscs and crustaceans (Bjomdal, 1997; Hatase, Omuta, & Tsukamoto, 2007). Typically, neritic stage turtles have smaller home ranges than those in oceanic habitats and they feed at relatively shallow depths (Schofield et al., 2010; Snape et al., 2016). Because of the availability of coasts surrounding the Tyrrhenian Sea, switching between oceanic and neritic foraging could enhance foraging opportunities, especially for the individuals diving on the Tunisian shelf and nearby the northern Sicilian coast. Here, only two turtles frequented known neritic foraging habitats (i.e., the large Tunisian plateau and the Amvrakikos Gulf), and once they started to use these areas, they did not return to the oceanic area before the tracking period was completed. The other turtles remained engaged in foraging over deep offshore waters and used shallow coastal waters mainly for transit. The difference in foraging movements can be seen when looking at the locations of high‐intensity diving classified by the HMM: within 30–60 km from the coast (Figure 6), between 20–40 km for #165767 diving on the Tunisian shelf, and farther away at 140 km (which is close to the maximum distance to the coast in the Tyrrhenian Sea). Such behavioral plasticity has been documented in adult loggerhead turtles (Hawkes et al., 2006) as well as juveniles (Mansfield et al., 2009). Our sample contained a large size range of juvenile turtles captured around an archipelago that is surrounded by deep water. We cannot be certain of which developmental stage these turtles were, and whether they had already chosen one foraging strategy over another (Howell et al., 2010). They may have been part of a mixed foraging aggregation consisting of oceanic stage turtles, juveniles in the transitional phase, and adults (i.e., the larger individuals) opportunistically foraging in the open sea. Indeed, a recent study on juvenile turtles captured in the same area as here suggested that these turtles preferentially feed on pelagic prey in oceanic habitats and then, as they reach a larger size, gradually enter neritic waters including in their diet more complex prey sources (Blasi, Tomassini, Gelippi, Insacco, & Polunin, 2018). The oceanic waters most frequented in the eastern Tyrrhenian Sea during their high‐intensity diving are consistent with those identified as a possible key foraging area for adult turtles of the same species, lending further support to the importance of this dynamic open sea habitat (Luschi et al., 2018).

Oceanic features such as currents, fronts, and eddies enhance primary productivity and aggregate zooplankton (Genin, Jaffe, Reef, Richter, & Franks, 2005; Yoder, Ackleson, Barber, Flament, & Balch, 1994), promoting foraging conditions that attract top predators, including cetaceans, sea turtles, pinnipeds, and seabirds (Cotté et al., 2011; Della Penna, De Monte, Kestenare, Guinet, & D'Ovidio, 2015; Scales et al., 2014, 2015; Kai et al., 2009). Features of the environment that promote prey occurrence in the top part of the water column are likely to drive foraging movements by near surface‐feeding marine predators (Boyd et al., 2015). Indeed, also loggerhead turtles have been found to associate with mesoscale oceanographic features (Howell et al., 2010; Kobayashi et al., 2011; Revelles et al., 2007). By switching from transit to low‐intensity diving and then to high‐intensity diving (Table 2), sea turtles engage in hierarchical foraging tactics, probably maximizing their chance of encountering aggregations of prey across patchy landscapes.

The Tyrrhenian Sea is a deep basin with complex bathymetry in which surface waters of Atlantic origin and salty intermediate waters coming from the eastern Mediterranean Sea get transformed and mixed (Iacono, Napolitano, Marullo, Artale, & Vetrano, 2013). It is one of the deepest basins in the Mediterranean, and robust, albeit seasonally changing, cyclonic and anticyclonic structures have been identified in correspondence to the main seamounts, the Vavilov (39.858N, 12.588E) and the Marsili (39.288N, 14.48E) in the eastern part. These dynamic features are also responsible for transporting nutrients and aggregating planktonic organisms and hence are likely good places for turtles to search for food. According to the marginal value theorem (MVT, Charnov (1976)), if patches vary in quality (profitability), a predator should leave the patch when the marginal capture rate falls to the average rate for the habitat. As the animal forages in the patch, the availability of food in the patch diminishes. Once rates of food gain drop, turtles have higher probabilities of switching from high‐intensity diving activities to low‐intensity diving activities, until the decision to leave the area by switching to transit is made (Table 4).

Large‐scale environmental features enhancing vertical and horizontal prey aggregations, as those described above, can be quite predictable (Embling et al., 2012). However, we lack an understanding of how fine‐scale spatial and temporal variation in size, intensity, and persistence of foraging patches are identified and how individuals find them. By incorporating additional sensors, bio‐logging tags can provide information on prey density, prey capture events, and high‐resolution environmental data, in fact revolutionizing the way in which the marine environment is monitored (Cox et al., 2017). Concurrent high‐resolution measurements of both habitat features and animal movements have a great potential but are still rare, especially in marine systems, and might present gaps in the recordings (Cox, Embling, Hosegood, Votier, & Ingram, 2018; March, Boehme, Tintoré, Vélez‐Belchi, & Godley, 2019). In our study, the environmental variable temperature was summarized at 6‐hr intervals since we aimed to highlight the spatial location of most used areas. The analysis showed that warmer water temperatures motivated juvenile sea turtles to further explore the area by engaging in a series of dives (Figure 7).

Since we had to group data at 6‐hr intervals, it was not possible to use these variables to answer questions on fine‐scale behavioral patterns. Novel hierarchical hidden Markov models and in‐depth analysis of underwater movements in relation to temperature and other ancillary environmental recordings (e.g., chlorophyll) will start clarifying underwater animal decision processes (Adam et al., 2019; Guinet et al., 2014; Leos‐Barajas et al., 2017). Both environmental data collected using animal‐borne tags as well as habitat availability are essential for these purposes. A more integrated and sustainable observing system (OOS) is required to facilitate environmental monitoring (March et al., 2019).

## 5 CONCLUSIONS

Highly mobile species might show different movements, behaviors, and habitat use in different life history stages. In our study, we have shown that smaller seas, as the Mediterranean Sea, characterized by both oceanic and neritic habitats in close proximity, host important habitats shared by loggerhead turtles of different life stages. More importantly, juvenile and adult loggerhead turtles share a comparatively small oceanic foraging area in the Southern Tyrrhenian Sea (this study and Luschi et al., 2018) that is characterized by fairly predictable oceanographic mesoscale features and hence making it a good candidate for ocean conservation. Only two individuals switched to neritic habitats, highlighting, once again, the importance of characterizing how, where, and when ontogenetic habitat shifts occur, especially in confined oceanic areas. Collecting high‐resolution information on individuals' behavior and distributions across different spatio‐temporal scales and life stages, as well as their interaction with the surrounding environment, is still challenging for marine ecosystems but important for the development of conservation and management actions (Hays et al., 2019).

Inferring population‐level dynamics (as survival and distribution) is also very challenging and related to the number of individuals tracked. The information obtained from HMMs about animals' behavior, distribution, activity budgets, and interaction with surrounding habitats can be further used. Approaches as habitat selection models, individual‐based models, and dynamic energy budget models can capitalize on such information and facilitate integration of data at both individual level and population level (Dalleau et al., 2019; Nabe‐Nielsen, Tougaard, Teilmann, Lucke, & Forchhammer, 2013; Sibly et al., 2013). By combining high‐resolution movement data, environmental data as well as knowledge about populations' status and dynamics in such novel modeling approaches, it will be possible to implement conservation policy and habitat management and to understand impacts of changing environment and anthropogenic activities on wild populations (Nabe‐Nielsen et al., 2018; Patterson et al., 2016; Pirotta, Edwards, New, & Thompson, 2018). Adaptive approaches as the dynamic ocean management (DOM) (Maxwell et al., 2015) and, importantly, multidisciplinary monitoring approaches across multiple spatio‐temporal scales are key to fill knowledge gaps and implement conservation management strategies. In this context, our study showed that the Tyrrhenian Sea could be a good place to start with implementing conservation measures in foraging areas that are urgently needed for the Mediterranean loggerhead turtle.

## CONFLICT OF INTEREST

None declared.

## AUTHOR CONTRIBUTIONS

**Marianna Chimienti:** Conceptualization (equal); data curation (equal); formal analysis (lead); methodology (equal); software (equal); validation (lead); writing–original draft (equal); writing–review and editing (equal). **Monica F. Blasi:** Conceptualization (equal); investigation (equal); methodology (equal); resources (supporting); writing–original draft (supporting); writing–review and editing (supporting). **Sandra Hochscheid:** Conceptualization (equal); data curation (equal); formal analysis (supporting); funding acquisition (lead); investigation (equal); methodology (equal); supervision (lead); validation (supporting); writing–original draft (equal); writing–review and editing (equal).

## Data Availability

The datasets generated during and/or analyzed during the current study are available in the Movebank Data Repository, https://doi.org/10.5441/001/1.1f1h87r8, ([Hochscheid,2020]).
